# SEAFEC: a spatial–edge adaptive convolution for multi-scale and boundary-aware plant disease and weed imagery

**DOI:** 10.3389/fpls.2025.1695076

**Published:** 2026-01-07

**Authors:** Cuimin Sun, Ji Liu, Biao He, Liuxue Huang, Lilan Lv

**Affiliations:** 1 School of Computer, Electronics and Information, Guangxi University, Nanning, China; 2 State Key Laboratory for Conservation and Utilization of Subtropical Agro-bioresources, Guangxi University, Nanning, China; 3 Guangxi Subtropical Crops Research Institute; Laboratory of Quality Risk Assessment for Agro-products of Ministry of Agriculture and Rural Affairs (Nanning); Key Laboratory of Quality and Safety Control for Subtropical Fruit and Vegetable, Ministry of Agriculture and Rural Affairs; Quality and Testing Center of Subtropical Fruit and Vegetable of Ministry of Agriculture and Rural Affairs, Nanning, Guangxi, China

**Keywords:** crop protection, convolutional neural networks (CNNs), boundary-aware feature modeling, smart agriculture, multi-scale representation data structure

## Abstract

**Introduction:**

Plant diseases and weeds are among the leading biological threats to global crop production. While deep learning has advanced automated analysis, existing approaches often fail under challenges like large multi-scale variations and blurred boundaries.

**Methods:**

To address this, we propose SEAFEC (Spatial-Edge Adaptive Feature Enhancement Convolution), a novel convolutional module that jointly enhances scale adaptivity and boundary precision. SEAFEC employs a dual-branch design: the SCARF branch dynamically adjusts receptive fields, while the MEFE branch explicitly strengthens edge features.

**Results:**

Across three representative tasks—plant disease classification, corn leaf disease detection, and sugarcane-weed segmentation—SEAFEC achieved consistent improvements (+1.8% accuracy, +2.5% mAP, +3.4% mIoU), with notable gains in boundary-sensitive cases.

**Discussion:**

These results highlight SEAFEC as a general-purpose enhancement module, providing a unified solution for tackling scale-boundary challenges in agricultural imagery to support reliable disease diagnosis and precision weed management.

## 1 Introduction

Agriculture plays a vital role in global food security, yet crop production is persistently threatened by weeds and plant diseases. Weeds compete with crops for nutrients, water, and light, causing yield losses of up to 30–40%, with severe infestations reducing yields by more than 50% (Oerke, 2006). Similarly, plant diseases remain a major constraint to agricultural productivity and quality worldwide, with the Food and Agriculture Organization (FAO) estimating 10–16% of global harvest losses annually, equivalent to hundreds of millions of tons of food (Food and Agriculture Organization of the United Nations, 2021). These threats highlight the urgent need for accurate and effective monitoring strategies to support precision agriculture, reducing excessive pesticide dependence and promoting sustainable crop protection.

With the rapid development of computer vision (CV) and deep learning, intelligent approaches have emerged as promising solutions for plant protection. Compared with traditional manual scouting, CV-based systems are non-destructive, scalable, and capable of real-time analysis in the field. Deep learning models have been applied to a wide range of agricultural tasks, including plant disease classification, lesion detection, and weed–crop segmentation (Kamilaris and Prenafeta-Boldú, 2018), achieving significant progress in crop monitoring and management.

Plant disease and weed imagery present diverse challenges in modern agriculture, and different computer vision strategies are typically adopted depending on the task requirements. For plant diseases, classification and object detection are the two most common approaches. In classification, the goal is to determine the disease type from leaf images, which is essential for early diagnosis and timely intervention. Early studies highlighted the strong feature extraction capability of deep networks, where convolutional neural networks (CNNs) (Lin et al., 2014; Szegedy et al., 2014) such as AlexNet (Krizhevsky et al., 2017), VGG (Simonyan and Zisserman, 2015), and ResNet (He et al., 2015) were applied to relatively clean datasets for end-to-end classification, significantly outperforming traditional machine learning methods. Beyond classification, object detection aims to localize disease lesions under complex field conditions, enabling severity assessment and guiding treatment decisions. This task has been widely addressed using frameworks such as Faster R-CNN (Ren et al., 2015), SSD (Liu et al., 2016), and YOLO (Redmon et al., 2016), with one-stage detectors like YOLOv5 (Ultralytics, 2022)and YOLOv8 (Ultralytics, 2023) proving particularly suitable for large-scale crops such as maize and rice due to their balance of accuracy and real-time efficiency.

For weeds, semantic segmentation is the preferred solution, as fine-grained pixel-level classification is often required for precision weeding. Accurate delineation of weed contours supports targeted removal and reduces crop competition. Fully convolutional networks (FCNs) (Long et al., 2015), including U-Net (Ronneberger et al., 2015), SegNet (Badrinarayanan et al., 2017), and DeepLab (Chen et al., 2018), dominate this domain by leveraging encoder–decoder structures with skip connections to integrate high-level semantics with low-level details, thereby achieving precise segmentation of irregular vegetation. However, existing approaches are often specialized for a single task, whereas agricultural imagery requires a general solution that can adapt across classification, detection, and segmentation.

Nevertheless, agricultural imagery poses unique challenges that limit the reliability of current approaches. In particular, multi-scale variations and blurred boundaries are ubiquitous: disease lesions may range from tiny spots to large irregular patches, often merging with healthy tissues; weeds appear as both sparse individuals and dense clusters, frequently overlapping with crop leaves. These characteristics make precise recognition difficult, and conventional convolutional operators with fixed receptive fields often fail to capture both large-scale context and fine-grained boundary details simultaneously. As a result, existing models are prone to misclassification, inaccurate lesion localization, or blurred segmentation.

To address these challenges, researchers have explored improved convolutional operators such as Deformable Convolutional Networks (DCNs) (Zhu et al., 2019), Dynamic Convolution (Chen et al., 2020), and RFAConv (Zhang et al., 2024). While these methods enhance receptive field flexibility, they often neglect explicit boundary refinement or overlook channel-wise interactions that are critical for distinguishing subtle disease symptoms. More importantly, they lack dedicated consideration of the scale–boundary dilemma that is intrinsic to agricultural images.

In this study, we propose the Spatial–Edge Adaptive Feature Enhancement Convolution (SEAFEC), a unified and highly effective convolutional structure specifically designed to address the dual challenges of scale diversity and boundary ambiguity in plant disease and weed imagery. SEAFEC adopts a dual-branch architecture: one branch dynamically adjusts receptive fields through spatial–channel attention to capture objects of varying scales and shapes, while the other explicitly enhances multi-scale edge features for sharper boundary localization. By integrating cross-channel interactions, SEAFEC jointly models semantic and structural information. As a result, it provides a robust solution that can be seamlessly applied to plant disease classification, lesion detection, and weed segmentation, thereby supporting more reliable agricultural monitoring.

## 2 Materials and methods

### 2.1 SEAFEC

Plant disease and weed images often display large variations in object scales and ambiguous boundaries, which remain major obstacles for accurate feature representation in agricultural vision. To address these challenges, we propose SEAFEC (Spatial–Edge Adaptive Feature Enhancement Convolution), an efficient plug-and-play module designed to improve both scale adaptivity and boundary precision.

**Figure 1:**
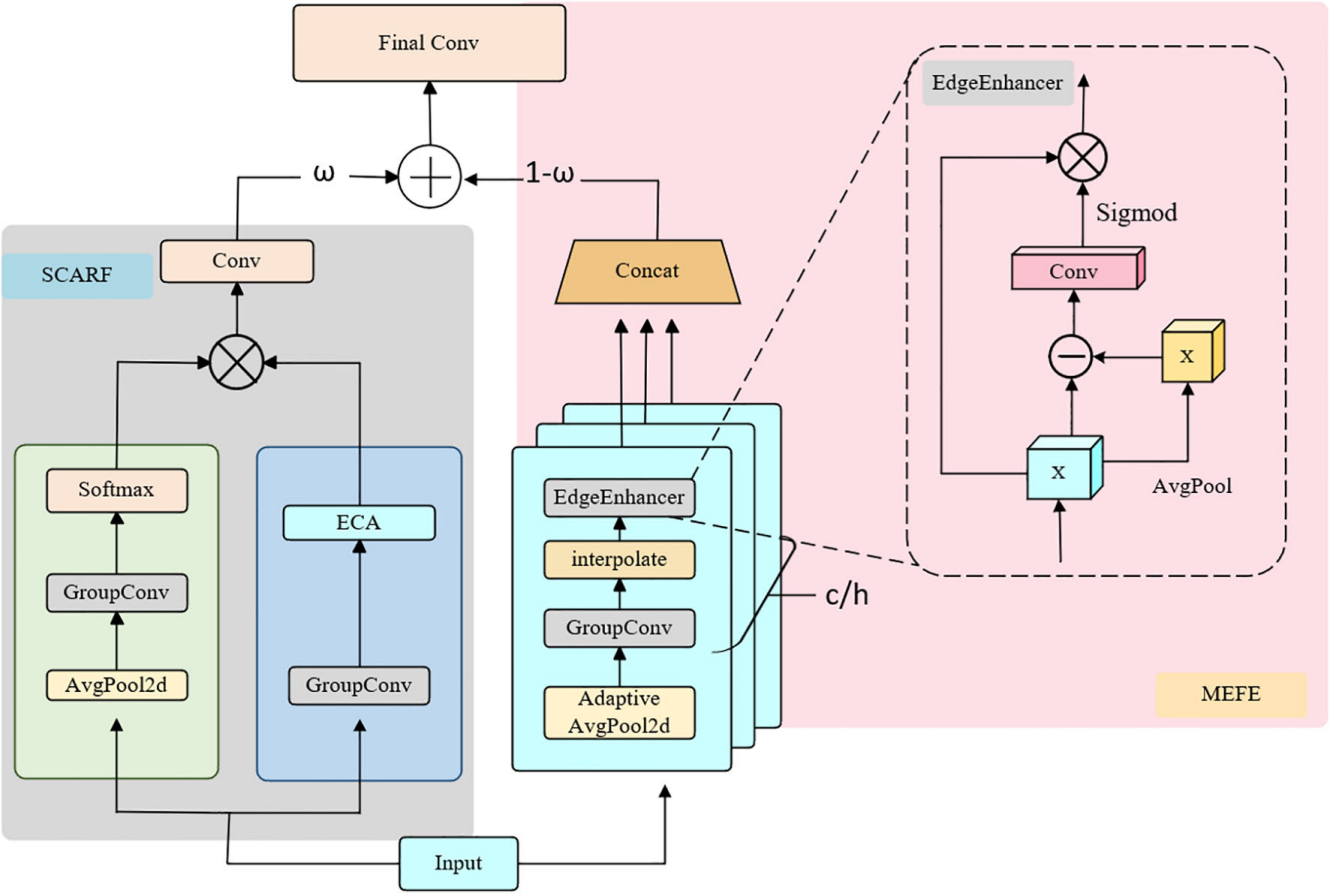
Structural diagram of the SEAFEC.


As illustrated in **Figure 1**, SEAFEC adopts a dual-branch architecture. The first branch, SCARF, dynamically adjusts receptive fields to capture lesions and weeds of varying sizes and irregular shapes. The second branch, MEFE, explicitly enhances edge and texture cues through learnable difference-based operations, thereby improving the delineation of blurred or overlapping boundaries. The outputs of the two branches are then adaptively fused to generate enhanced representations that integrate both structural and boundary information.

$${\text{F}}_{\mathrm{out}}=\text{Fuse}\left(\text{SCARF}\left({\text{F}}_{\mathrm{in}}\right),\text{MEFE}\left({\text{F}}_{\mathrm{in}}\right)\right)$$

This design enables SEAFEC to better capture the multi-scale variability and fine-grained edge details that are critical in plant disease classification, disease detection, and weed segmentation.

### 2.2 SCARF

**Figure 2:**
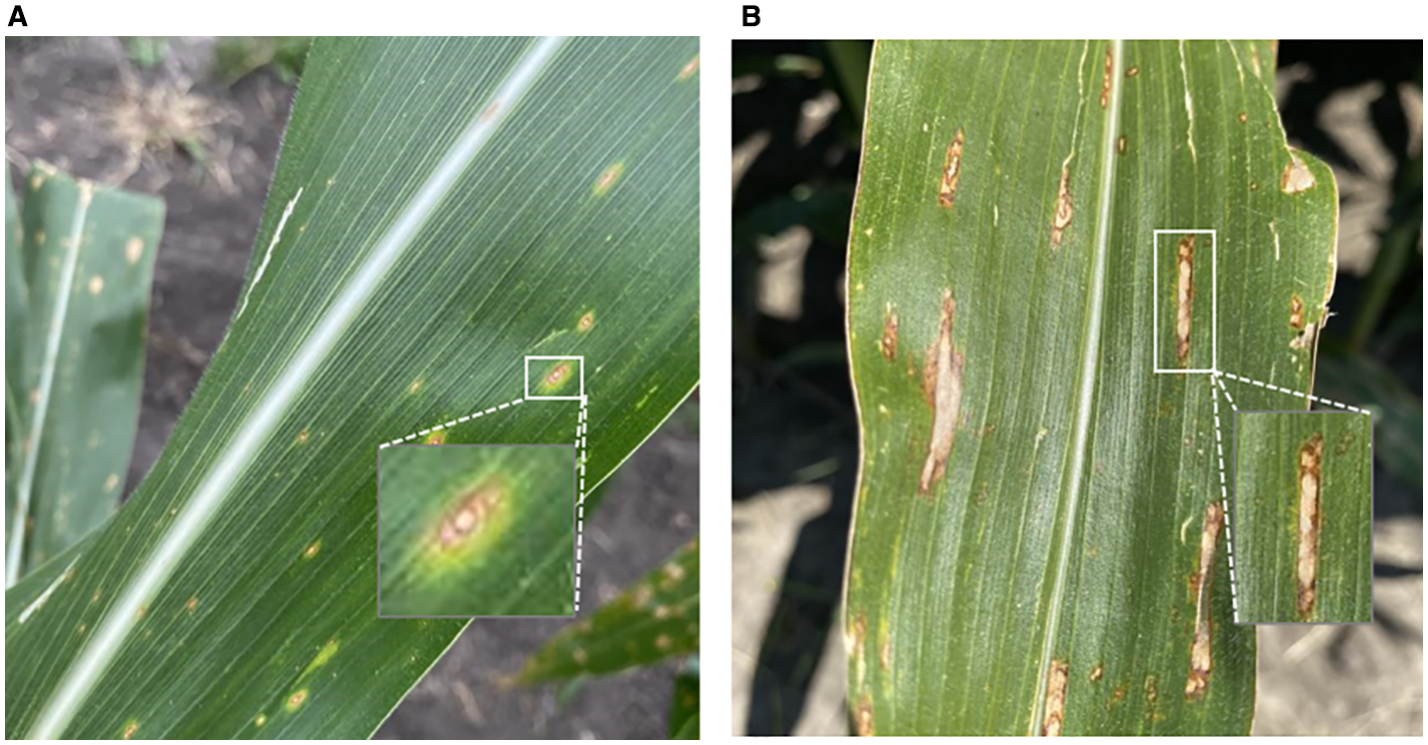
Illustration of morphological and scale differences between two typical corn leaf diseases. **(a)** Northern Leaf Spot: lesions appear as dispersed, near-circular spots. **(b)** Northern Leaf Blight: lesions spread along the veins, forming elongated and irregular strip patterns. This figure highlights the high variability in shape and scale of plant diseases in agricultural imagery.

In plant disease and weed image analysis, scale variation and irregular morphology remain major obstacles for reliable feature extraction. As illustrated in **Figure 2**, maize leaf lesions may range from tiny circular spots to elongated strip-like areas, while weed objects often exhibit diverse shapes under occlusion and illumination. Conventional convolutions, with their fixed receptive fields, cannot simultaneously capture such heterogeneous patterns, leading to loss of critical lesion details or redundant background features.

**Figure 3:**
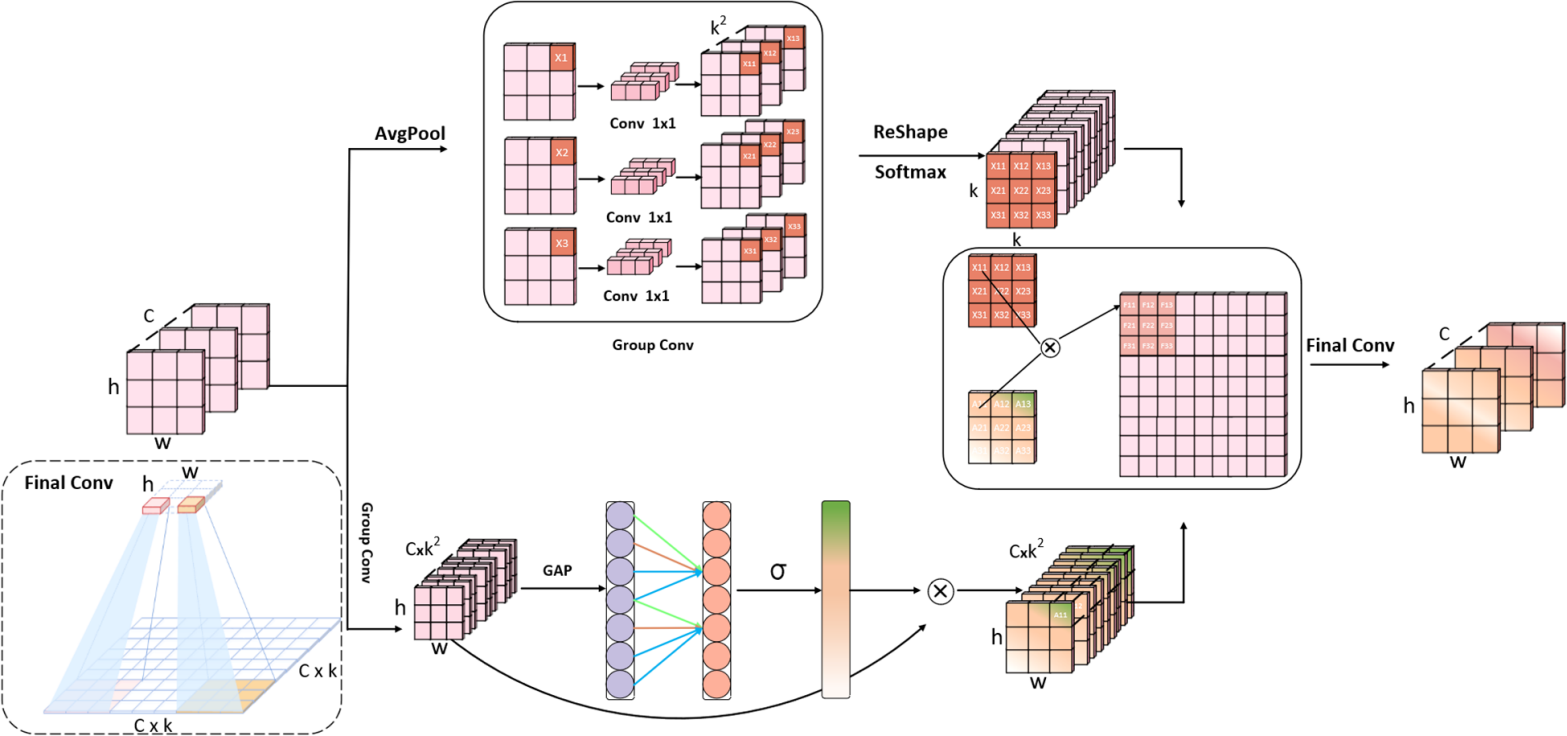
Structural diagram of the SCARF module.

To overcome this, we design the Spatial–Channel Adaptive Receptive Field Attention (SCARF) module. The overall architecture of the proposed SCARF module is illustrated in **Figure 3**. The core idea is to replace the static convolution kernel with a dynamic, input-dependent process, enabling receptive fields to adjust adaptively to target scales and shapes. Unlike computationally expensive global self-attention, SCARF retains the local inductive bias of convolution while introducing a lightweight attention branch. This branch generates position-specific convolutional weights for each spatial location, while a channel attention mechanism further strengthens inter-channel semantic interactions. In this way, SCARF provides both spatial adaptivity and channel awareness, which are crucial for distinguishing disease symptoms that differ subtly in color and texture.

Formally, given an input feature map $X\in R^{B\times C\times H\times W}$, SCARF first extracts contextual representations by average pooling, followed by a 1×1 group convolution that expands the channel dimension from C to $C\times k^{2}$, where k  represents the kernel size that determines the receptive field extent. The resulting attention weights are normalized with a Softmax function to obtain the spatial attention tensor.


$${\text{A}}_{\mathrm{rf}}=\text{Softmax}\left({\text{GroupConv}}_{\text{C}\to \text{C}\times {\text{k}}^{2}}^{1\times 1}\left(\text{AvgPool}\left(\text{X}\right)\right)\right)$$

where $A_{rf}\in R^{B\times C\times k^{2}\times H\times W}$ encodes the relative importance of each receptive field location. In parallel, multi-receptive-field features are extracted through a k ×k group convolution, batch normalization, and non-linear activation. Since color cues play a crucial role in distinguishing different plant diseases (e.g., yellow rust vs. powdery mildew) and separating weeds from crops, we further incorporate channel-wise recalibration. To this end, an Efficient Channel Attention (ECA) (Wang et al., 2020) module is applied to emphasize disease- and weed-relevant channels while suppressing irrelevant responses:


$${\text{F}}_{\mathrm{rf}}=\text{ECA}\left(\sigma \left(\text{BN}\left({\text{GroupConv}}_{\text{C}\to \text{C}\times {\text{k}}^{2}}^{\text{k}\times \text{k}}\left(X\right)\right)\right)\right)$$

producing feature maps that emphasize disease-relevant channels while suppressing noise.

Finally, the attention tensor $A_{rf}$ modulates these features by element-wise multiplication, yielding weighted responses:


$${\text{F}}_{\text{weighted}}={\text{A}}_{\mathrm{rf}}⊙{\text{F}}_{\mathrm{rf}},$$

which are rearranged and fused by a stride-k convolution to restore the original resolution:


$$\text{Y}={\text{Conv}}_{\text{k}\times \text{k},\text{stride}=\text{k}}\left(\text{Rearrange}\left({\text{F}}_{\text{weighted}}\right)\right),$$

where $Y\in R^{B\times C\times H\times W}$ is the final output.

Through this design, SCARF achieves adaptive context aggregation while remaining computationally efficient, making it suitable for backbone integration in agricultural disease recognition and weed detection tasks.

### 2.3 MEFE

**Figure 4:**
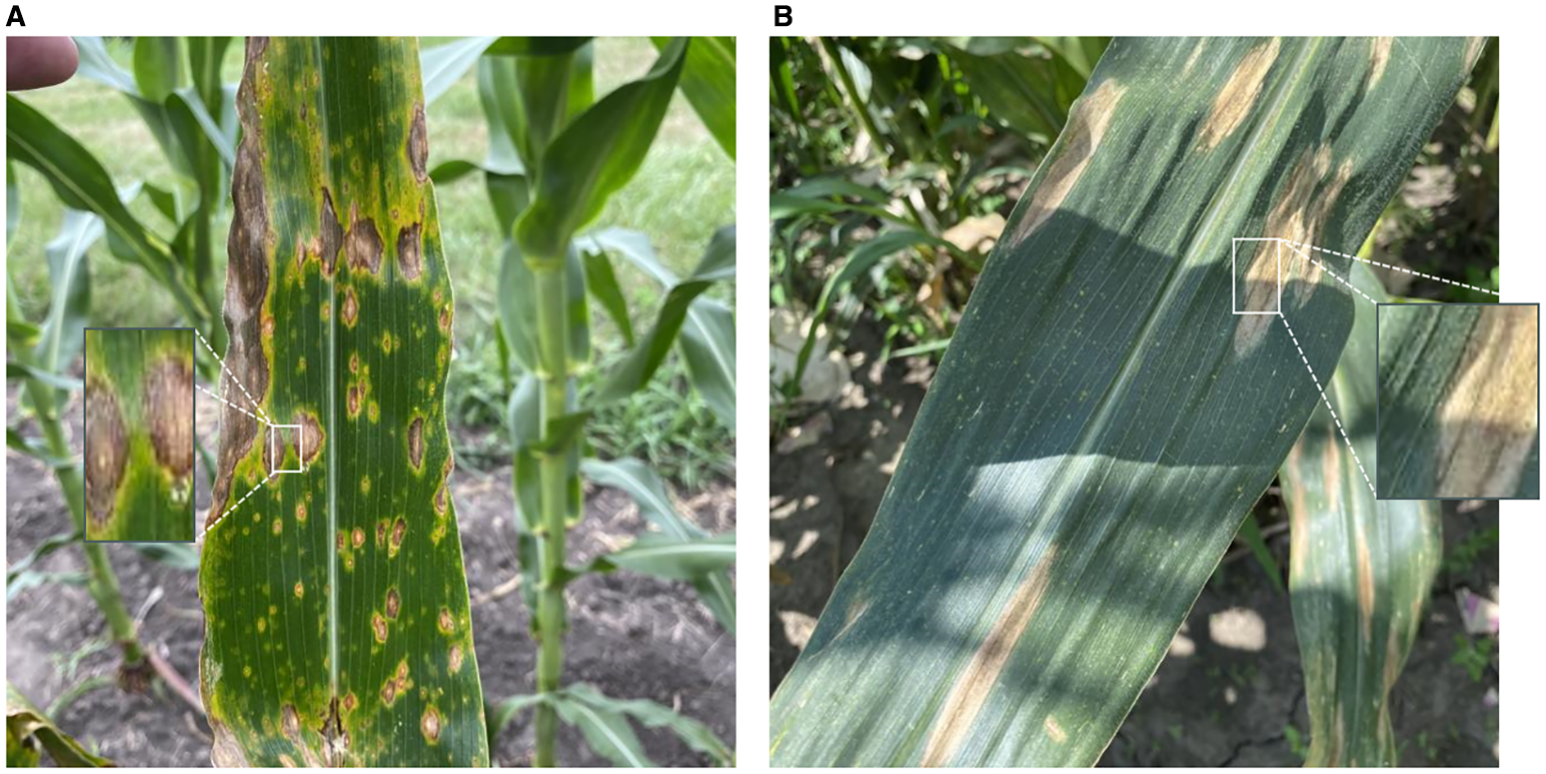
Visual challenges in disease recognition under natural field conditions. **(a)** Coexistence of multiple disease types with blurry transitional halos along lesion boundaries, making it difficult to distinguish from healthy tissue; **(b)** Severe lighting variations, including shadow occlusion and strong reflections, further degrade the visibility of edge features. This figure illustrates typical perceptual challenges such as boundary ambiguity and texture interference in agricultural imagery.

Although the SCARF module enables adaptive receptive field adjustment based on semantic cues, it is still limited in explicitly capturing boundary-local texture details. In real agricultural imagery, this limitation becomes particularly critical. For instance, lesion boundaries often blend with healthy tissues, forming blurred halos that hinder precise localization, while weeds and crops frequently overlap with jagged and intertwined contours under natural field conditions. Moreover, lighting variations such as shadows or reflections further degrade boundary visibility, making conventional convolutions insufficient for reliable delineation. These typical challenges are illustrated in **Figure 4**, which shows how boundary ambiguity and texture interference can significantly affect disease recognition under field conditions.

**Figure 5:**
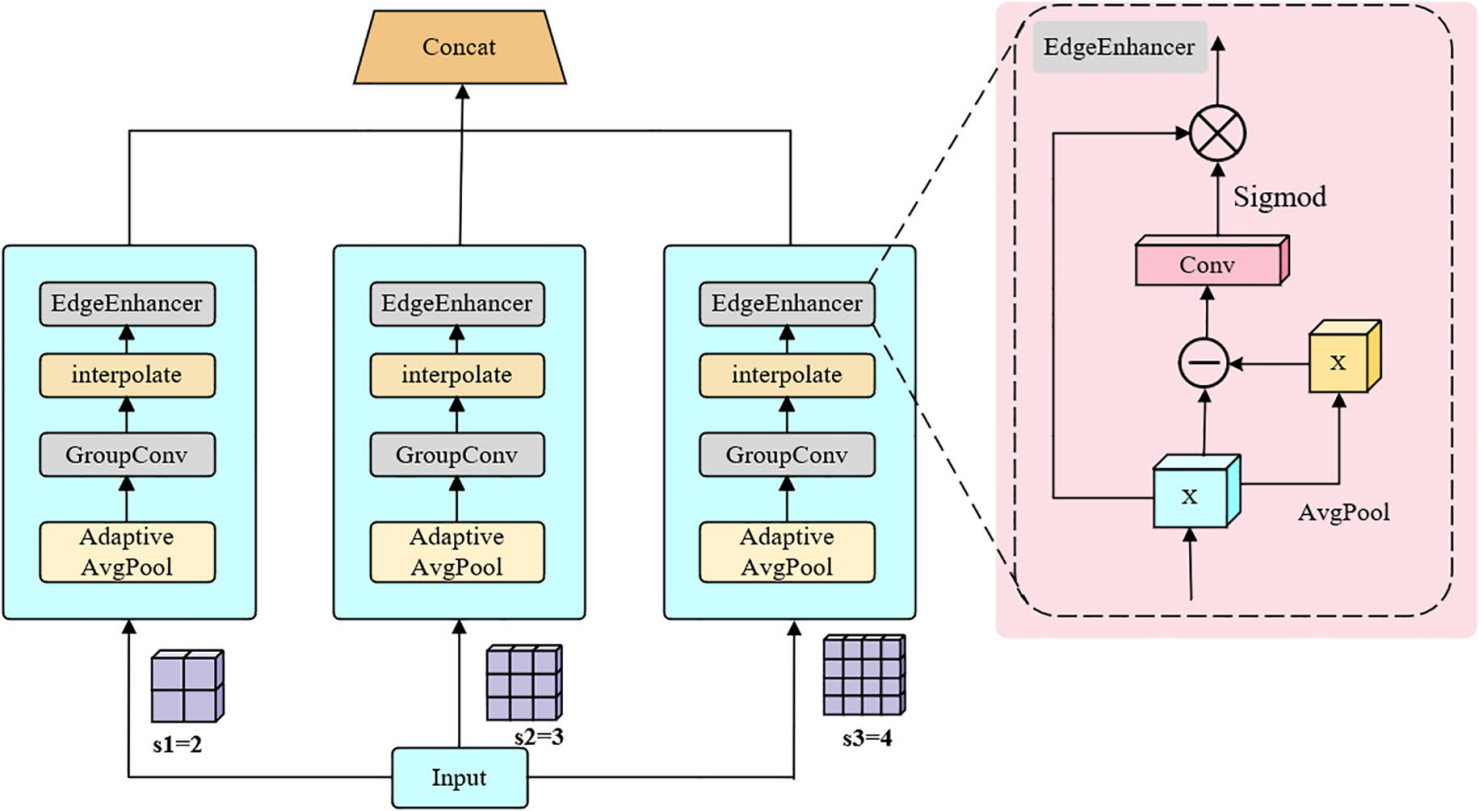
Structural diagram of the MEFE module.

To address this issue, we design the Multi-Scale Edge Feature Enhancement (MEFE) module, which explicitly strengthens edge and texture cues in a lightweight and self-supervised manner. Unlike traditional edge operators (e.g., Sobel, Laplacian) that are fixed and non-trainable, MEFE introduces learnable difference-based operations across multiple scales, enabling robust enhancement of both fine and coarse boundaries without requiring extra edge annotations. The overall structure of MEFE is shown in **Figure 5**.

Formally, given an input feature map $X\in R^{B\times C\times H\times W}$, MEFE first applies adaptive average pooling with multiple kernel sizes to obtain contextual features at different scales. Each branch performs a 1 ×1 convolution for channel reduction, followed by a depthwise separable convolution to capture local spatial variations. The extracted multi-scale features are then upsampled to the original resolution:


$${\text{F}}_{i}=\text{DSConv}\left({\text{Conv}}_{1\times 1}\left({\text{Pool}}_{\mathrm{si}}\left(X\right)\right)\right),$$

Where: ${\text{Pool}}_{s_{i}}$ denotes adaptive pooling with kernel size $S_{i}.$Next, each feature map $F_{i}$ undergoes an edge enhancement step that mimics a data-driven high-pass filter. Specifically, a smoothed version of $F_{i}$ obtained by average pooling is subtracted from the original feature, yielding a high-frequency edge response $E_{i}$:


$${\text{E}}_{\text{i}}={\text{F}}_{\text{i}}-{\text{AvgPool}}_{3\times 3}\left({\text{F}}_{\text{i}}\right).$$

This response is further refined by a lightweight convolutional subnetwork with residual connections, ensuring stable gradient flow and improved texture modeling:


$$\hat{{\text{F}}_{\text{i}}}={\text{F}}_{\text{i}}+\phi \left({\text{E}}_{\text{i}}\right),$$

where ϕ(·)  denotes the refinement operation. Here, ϕ(·)  denotes a lightweight residual refinement subnetwork implemented with depthwise separable convolutions to enhance fine edge structures while maintaining computational efficiency.

Finally, all enhanced features $\hat{F_{i}}\;\;$ are concatenated along the channel dimension and fused by a 1 ×1 convolution to restore the channel size:


$$\text{Y}={\text{Conv}}_{1\times 1}\left(\left[\hat{{\text{F}}_{1}},\hat{{\text{F}}_{2}},\ldots ,\hat{{\text{F}}_{n}}\right]\right),$$

resulting in the output $Y\in R^{B\times C\times H\times W}.$By explicitly reinforcing edge cues across multiple scales, MEFE significantly improves the delineation of blurred lesion boundaries and intertwined weed–crop contours. This makes it particularly valuable for boundary-sensitive agricultural vision tasks, such as disease detection and weed segmentation, where fine structural details directly affect recognition accuracy and subsequent management decisions.

### 2.4 Integration into SEAFEC

While the SCARF module demonstrates strong adaptability in modeling scale diversity and irregular shapes, the MEFE module explicitly enhances the perception of edges and fine-grained textures. However, in real agricultural imagery, lesion regions and weed targets often exhibit both complexities simultaneously—large spatial extent with indistinct boundaries. This highlights the need for a unified design that can jointly address scale adaptivity and boundary enhancement.

To this end, we integrate SCARF and MEFE into the Spatial–Edge Adaptive Feature Enhancement Convolution (SEAFEC), as illustrated in **Figure 1**. Specifically, SCARF provides global receptive-field adjustment to capture multi-scale lesion and weed structures, while MEFE refines local edges to ensure precise delineation of disease spots and crop–weed boundaries.

The outputs of the two branches, $F_{\text{SCARF}}\text{ and }F_{\text{MEFE}}$, are combined through an attention-based fusion mechanism. Instead of simply concatenating or adding, we assign each branch a learnable weight α∈[0,1]  obtained via a Sigmoid activation, and compute the final output as: $Y=\alpha \cdot F_{\text{SCARF}}+\left(1-\alpha \right)\cdot F_{\text{MEFE}},$where $Y\in R^{B\times C\times H\times W}$ is the enhanced feature representation. This adaptive weighting enables SEAFEC to dynamically emphasize either scale–shape modeling or edge–texture enhancement, depending on the characteristics of the input image.

Through this dual-branch fusion, SEAFEC unifies dynamic receptive field adaptation and multi-scale edge enhancement within a single framework, significantly improving feature extraction for plant disease classification, corn disease detection, and weed segmentation.

To further highlight the high effectiveness and generality of SEAFEC in plant disease and weed management, we evaluate it on three complementary tasks with distinct methodological paradigms: disease classification, disease detection, and weed segmentation. This design not only verifies the broad applicability of SEAFEC across image-level, object-level, and pixel-level recognition, but also demonstrates its strong capability to deliver consistently superior performance under diverse conditions. At the same time, these tasks directly correspond to practical needs in crop protection: (i) disease classification enables rapid recognition of multiple disease types for early intervention, (ii) disease detection provides lesion localization for precise and timely treatment, and (iii) weed segmentation ensures accurate crop–weed boundary delineation to guide precision weeding. Through this comprehensive evaluation, SEAFEC is demonstrated to be a highly effective and versatile module capable of supporting both plant disease diagnosis and weed control, two essential pillars of modern smart agriculture.

## 3 Experiments and results

### 3.1 Experimental environment

All experiments were conducted on a system running Ubuntu 22.04.4 LTS, equipped with an Intel Core i9-14900K processor (32 cores), 128 GB of RAM, and an NVIDIA GeForce RTX 4090 GPU with 24 GB of memory. To ensure experimental controllability and reproducibility, all training and evaluation procedures were executed independently on a single GPU, without multi-GPU parallelism. The software environment consisted of Python 3.10, PyTorch 2.3.0, CUDA 12.1, and cuDNN version 8902. A detailed summary of the experimental configuration is provided in **Table 1**.

**Table 1:** Hardware and software configuration of the experimental environment.

Component	Specification
Operating System	Ubuntu 22.04.4 LTS
Processor (CPU)	Intel Core i9-14900K
Memory (RAM)	128 GB
GPU	NVIDIA RTX 4090
Python	3.10
PyTorch	2.3.0
CUDA	12.1

### 3.2 Plant disease classification experiments

To evaluate the capability of the proposed modules in plant disease recognition, we adopt the widely used PlantVillage dataset (Hughes and Salathé, 2015) as a benchmark for classification experiments. Disease classification serves as a fundamental step in plant health management, enabling rapid identification of pathogen types for early intervention. PlantVillage contains 60,343 leaf images covering 38 disease categories across 14 crop species, including representative cases such as apple scab, cherry powdery mildew, corn northern leaf blight, and grape black rot. The dataset is widely used in plant pathology research due to its clean backgrounds and clearly defined lesion regions, making it suitable for isolating and assessing the modules’ effectiveness before moving to more complex field imagery. For fair evaluation, the dataset is divided into training, validation, and testing sets in a 7:2:1 ratio, ensuring sufficient training samples while enabling reliable monitoring and objective assessment of model performance, and providing a solid baseline for verifying the general effectiveness of SEAFEC in disease recognition.

For the classification task, performance is evaluated using Accuracy, Precision, Recall, Specificity, and F1 Score. Accuracy measures overall correctness, Precision and Recall reflect the model’s reliability and ability to identify positive cases, Specificity indicates how well false positives are avoided, and F1 Score provides a balanced assessment, especially for imbalanced datasets.

The network was trained using the Adam optimizer with an initial learning rate of 1×10^-^³ and a batch size of 32. The learning rate was reduced by a factor of 0.1 when validation performance plateaued. The random seed was fixed at 42 for all experiments to ensure reproducibility.

In the proposed SEAFEC module, the fusion coefficient α was initialized to 0.6 and learned adaptively during training. The pooling kernel sizes in the MEFE branch were set to (Szegedy et al., 2014; Simonyan and Zisserman, 2015; Kamilaris and Prenafeta-Boldú, 2018) for the classification task.

We adopt ResNet-18 (He et al., 2015) as the backbone network and integrate the proposed modules into the first 3×3 convolution layer of each BasicBlock to enhance hierarchical feature representation, as shown in **Table 2**.

**Table 2:** Structural comparison between the original ResNet-18 and the modified versions.

Layer name	Output size	Original resNet-18	Our resNet-18
Conv1	112 × 112	Conv 7x7	
Layer1	56 × 56	$\left[\begin{array}{c} \;Conv\;3\times 3\\ \;\;Conv\;3\times 3\; \end{array}\right]$ × 2	$\left[\begin{array}{c} \;newConv\;3\times 3\\ \;\;Conv\;3\times 3\; \end{array}\right]$ × 2
Layer2	28 × 28	$\left[\begin{array}{c} \;Conv\;3\times 3\\ \;\;Conv\;3\times 3\; \end{array}\right]$ × 2	$\left[\begin{array}{c} \;newConv\;3\times 3\\ \;\;Conv\;3\times 3\; \end{array}\right]$ × 2
Layer3	14 × 14	$\left[\begin{array}{c} \;Conv\;3\times 3\\ \;\;Conv\;3\times 3\; \end{array}\right]$ × 2	$\left[\begin{array}{c} \;newConv\;3\times 3\\ \;\;Conv\;3\times 3\; \end{array}\right]$ × 2
Layer4	7 × 7	$\left[\begin{array}{c} \;Conv\;3\times 3\\ \;\;Conv\;3\times 3\; \end{array}\right]$ × 2	$\left[\begin{array}{c} \;newConv\;3\times 3\\ \;\;Conv\;3\times 3\; \end{array}\right]$ × 2
1 × 1			Average Pool		

As shown in **Table 3**, Experimental results on the test set demonstrate that the SCARF module, which focuses on adaptive receptive field modeling, yields a notable improvement in classification accuracy (+1.34%), outperforming the edge enhancement module MEFE (+0.64%). This suggests that for high-level semantic classification tasks, accurately capturing the global shape, distribution, and macro-texture of lesions is essential—an area where SCARF excels by dynamically adjusting its receptive field to attend to both early-stage small spots and large-scale lesions.

**Table 3:** Ablation results of different modules on the plant disease classification task.

Network	Accuracy(%)	Precision(%)	Recall(%)	Specificity (%)	F1Score(%)
ResNet-18	95.81	94.13	94.90	99.91	94.51
+MEFE	96.45	95.28	95.85	99.92	95.56
+SCARF	97.15	96.42	96.78	**99.94**	96.60
+SEAFEC	**97.85**	**97.32**	**97.15**	**99.94**	**97.23**

The bold values indicate the best performance (highest metric) in each column.

When both modules are integrated, the SEAFEC-enhanced network achieves the highest accuracy (97.85%), surpassing either submodule alone. This validates the complementary strengths of SCARF and MEFE in joint feature modeling. While classification relies heavily on global semantic information, the fine-grained boundary cues provided by MEFE remain critical in distinguishing disease types with similar overall appearance but subtle edge differences. The fusion mechanism in SEAFEC improves both model robustness and generalization ability.

**Figure 6:**
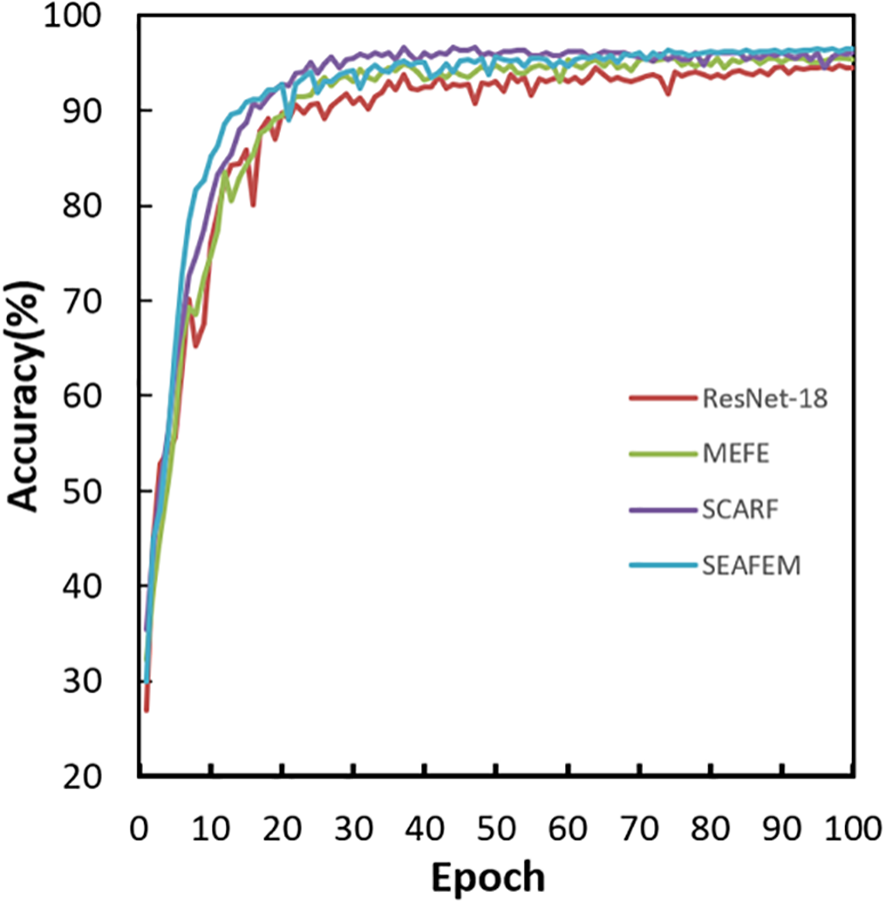
Performance comparison of different models during training.

Moreover, this performance advantage extends beyond final accuracy to the entire training process. As shown in **Figure 6**, models equipped with our modules converge faster and achieve more stable accuracy compared to the baseline. Notably, SCARF and SEAFEC exhibit rapid accuracy gains during the early training stages, indicating that their adaptive mechanisms enable the network to quickly focus on discriminative features, thereby accelerating the learning process.

As shown in **Table 4**, the baseline ResNet-18 contains 11.69M parameters. Introducing the SCARF module leads to a slight increase of +1.4% (11.85M), while the MEFE module significantly reduces parameters by 30.7% (8.10M). In contrast, the SEAFEC fusion design results in a substantial increase to 15.98M (+36.7%).

**Table 4:** Parameter and FLOPs variations of ResNet18 with different modules integrated.

Model	Params(M)	ΔParams(M)	FLOPs(G)	ΔFLOPs(G)
ResNet18	11.69	—	1.81	—
+SCARF	11.85	+1.4%	1.87	+3.3%
+MEFE	8.1	-30.70%	1.18	-34.80%
+SEAFEC	15.98	+36.7%	2.38	+31.5%

The bold values indicate the best performance (highest metric) in each column.

The parameter changes reflect the underlying design principles of each module. SCARF introduces only lightweight spatial and channel attention operations, adding a negligible increase in parameters while delivering clear accuracy gains—demonstrating its efficiency. MEFE achieves the largest reduction by replacing standard convolutions with multi-scale grouped depthwise convolutions, striking an excellent balance between compactness and feature representation. In contrast, SEA-FEC substantially increases parameters due to concatenation and multi-layer fusion after parallel processing, which doubles intermediate channel dimensions; however, this design prioritizes accuracy, showing that modest increases in computational cost can translate into significant improvements in feature modeling.

**Figure 7:**
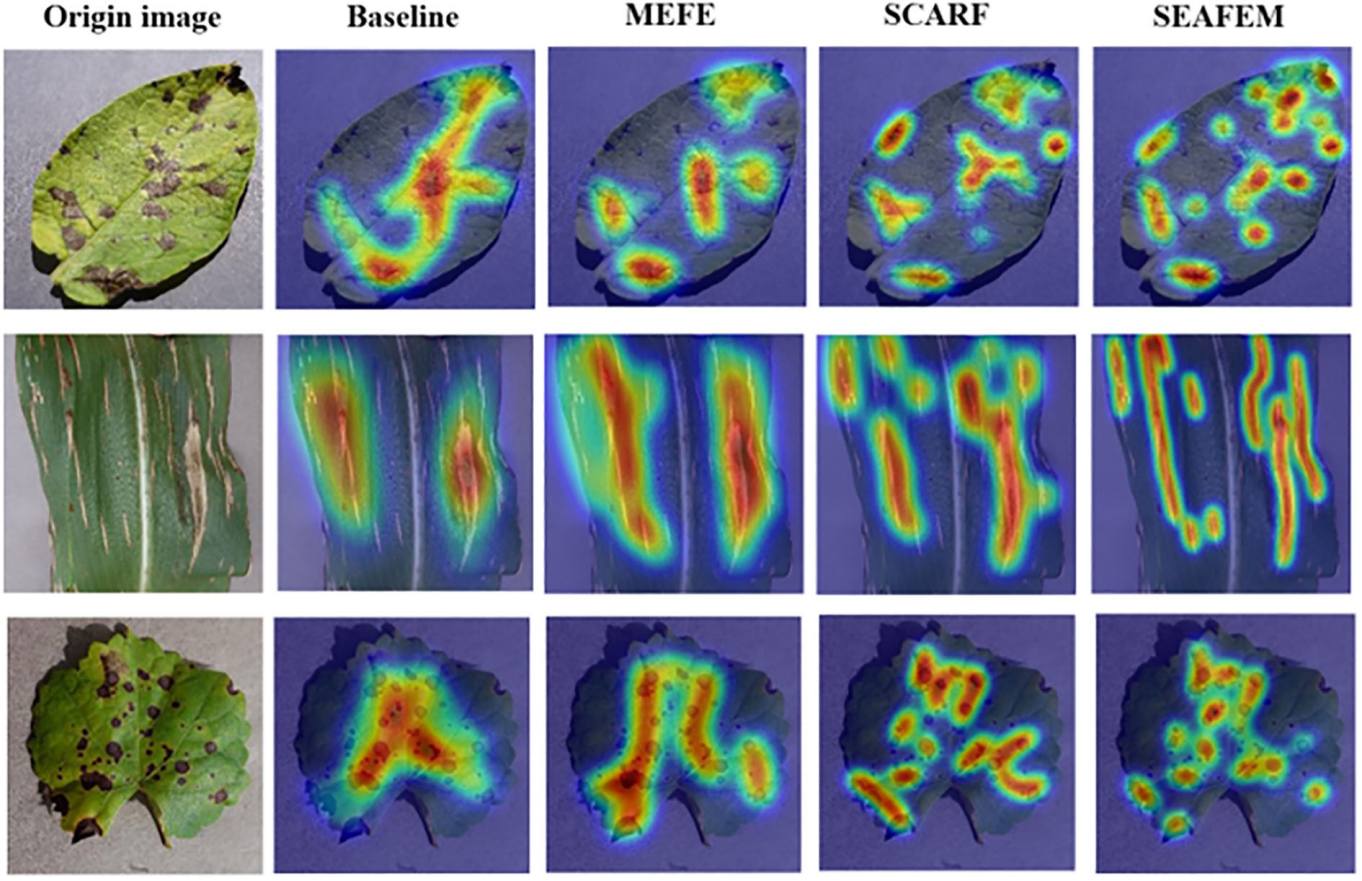
Visualization comparison of class activation maps (CAM) across different modules on the validation set. This figure illustrates how the baseline model and its variants integrated with the MEFE, SCARF, and SEAFEC modules attend to key regions in the image during classification.

This synergistic enhancement effect is further validated through class activation map (Grad-CAM) (Selvaraju et al., 2017) visualizations, as shown in **Figure 7**. Compared to the baseline model, SEAFEC produces attention heatmaps that not only better cover lesion regions of varying scales but also exhibit sharper and more focused activation boundaries—attributable to the edge enhancement provided by the MEFE module. These results indicate that SEAFEC effectively captures both the macro-level structural features and fine-grained edge details of the target, thereby improving the model’s discriminative capacity and representation quality in classification tasks.

### 3.3 Corn disease detection experiments

To further assess the effectiveness of the proposed modules in addressing real-world plant disease challenges, we applied them to the CD&S maize disease dataset (Ahmad et al., 2021), a benchmark specifically designed for object detection under complex field conditions. Unlike controlled datasets with clean backgrounds, CD&S images were captured directly in the field, often featuring cluttered backgrounds, variable illumination, and overlapping leaves, which make lesion localization particularly challenging. The dataset covers three major maize leaf diseases: Northern Leaf Blight (NLB), Gray Leaf Spot (GLS), and Northern Leaf Spot (NLS), with 511, 524, and 562 original images, respectively. To increase diversity and robustness, data augmentation techniques such as rotation, cropping, scaling, blurring, and noise addition were applied, doubling the number of images per class to 1022, 1048, and 1124, respectively. The enhanced dataset was then split into training, validation, and testing sets with a ratio of 7:2:1, allowing us to examine whether SEAFEC can maintain high effectiveness when moving from controlled environments to real-world field imagery.

Compared with the PlantVillage dataset, CD&S presents greater challenges for detection, as it contains samples of varying shapes and sizes across multiple growth stages. Moreover, many images include dense and small targets, making accurate detection and localization more difficult.

For the object detection task, performance is evaluated using mAP@50 (%) and mAP@50–95 (%). mAP@50 measures the mean average precision at an Intersection over Union (IoU) threshold of 0.5, while mAP@50–95 averages the precision over IoU thresholds from 0.5 to 0.95, providing a more comprehensive assessment of detection accuracy.

To select a suitable detection backbone, we first conducted a comprehensive horizontal evaluation of various lightweight YOLO-based models. As shown in **Table 5**, we compared multiple state-of-the-art YOLO variants in terms of accuracy and inference efficiency. Based on this comparison, we selected YOLOv11n (Kamilaris and Prenafeta-Boldú, 2018) as the primary baseline due to its optimal trade-off between precision and speed. Additionally, YOLOv8n (Lin et al., 2014), a widely adopted detector, was retained as a secondary baseline to ensure the generalizability and robustness of our findings.

**Table 5:** Performance and complexity comparison of different lightweight YOLO models.

Module	mAP@50 (%)	mAP@50-95(%)	Params(M)	FLOPs(G)
yolov8n	74.2	37.6	3.01	8.1
yolov10n	73.4	37.2	**2.27**	6.5
yolov11n	**74.8**	**37.7**	2.58	**6.3**
yolov12n	73.6	37.4	2.56	**6.3**

The bold values indicate the best performance (highest metric) in each column.

Following this selection, we established the training configuration for the baseline models, implemented in PyTorch. The models were trained for 300 epochs using the SGD optimizer with a momentum of 0.937, weight decay of 5×10^-4^, and an initial learning rate of 5×10^-^³. All experiments utilized an input resolution of 640×640 and a batch size of 16. To ensure reproducibility, a random seed of 42 was set with deterministic mode enabled.

In the SEAFEC-integrated variant, the fusion coefficient αwas initialized to 0.5 and optimized during training, while the MEFE pooling kernel sizes were set to (Simonyan and Zisserman, 2015; Redmon et al., 2016; Kamilaris and Prenafeta-Boldú, 2018) to better accommodate varying lesion sizes in the CD&S dataset.

Our proposed module was integrated into the C2f blocks of the YOLO backbone, aiming to enhance feature representation from the early stages of the network by strengthening spatial structure and boundary modeling.

**Figure 8:**
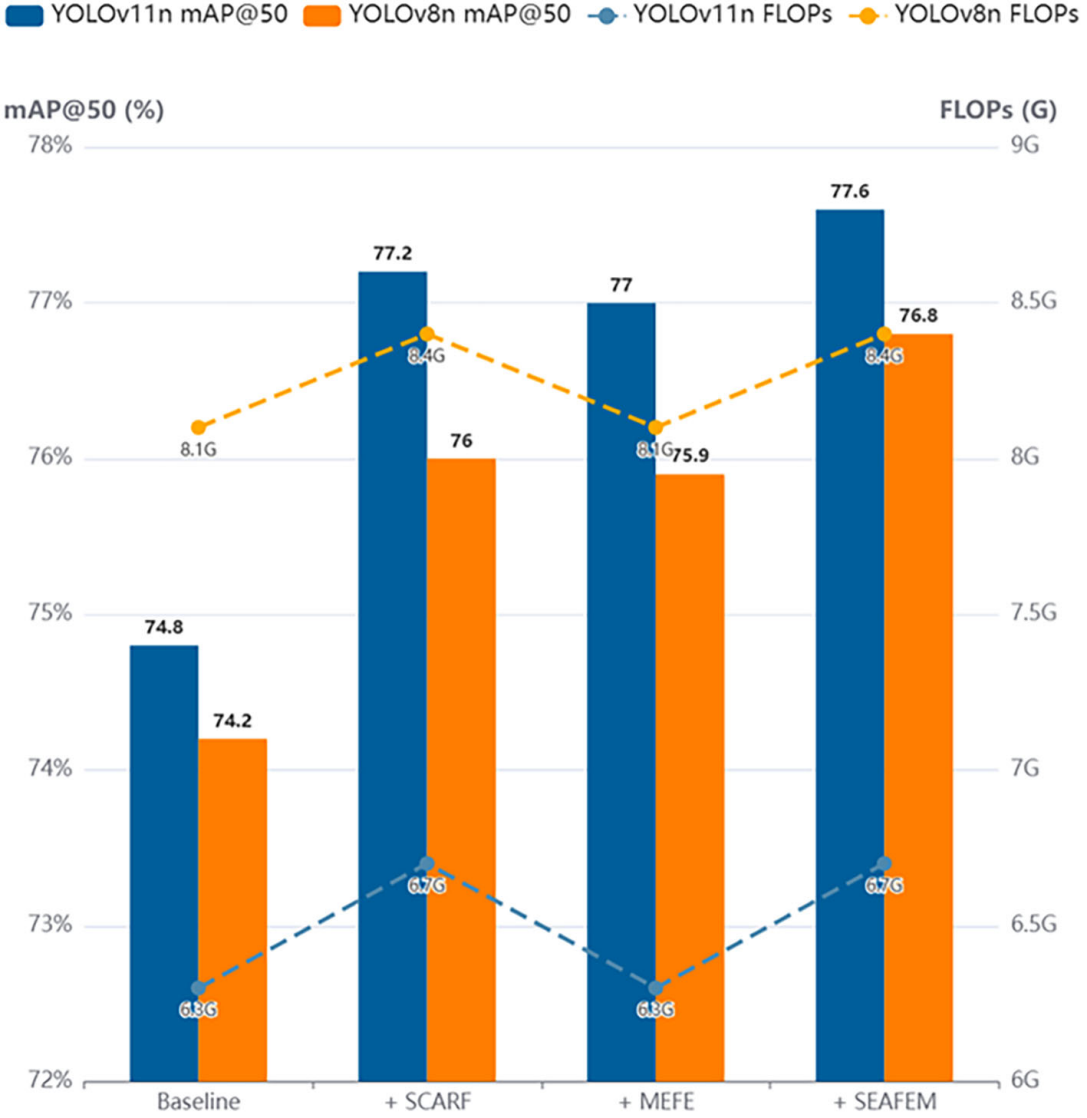
Experimental results of different modules on YOLOv11n and YOLOv8n.

As shown in **Figure 8**, unlike the classification task where SCARF exhibited dominant performance, the contributions of SCARF and MEFE become more balanced in object detection. This shift is attributed to the dual challenge posed by detection tasks, which require both accurate class prediction and precise bounding box regression. Specifically, SCARF enhances the model’s perception of targets at multiple scales through its adaptive receptive field mechanism, resulting in a 2.4-point increase in mAP@50 on YOLOv11n. Meanwhile, MEFE improves boundary localization by explicitly enhancing contour features, achieving a 2.2-point mAP gain without adding any computational cost and even slightly reducing the parameter count.

These results demonstrate that explicit edge enhancement is a highly efficient and low-cost strategy for improving detection performance—particularly valuable in lightweight models where computational resources are constrained.

Ultimately, the integrated SEAFEC module once again achieves the best detection performance, with a mAP@50 of 77.6%. Unlike the classification task, where the two modules played clearly differentiated roles, SCARF and MEFE exhibit equally important and complementary characteristics in object detection. SCARF improves the model’s global perception of targets at varying scales, leading to higher recall, while MEFE enhances localization accuracy by refining edge details. Their synergy enables the model to meet both core demands of detection: robust recognition and precise localization.

**Figure 9:**
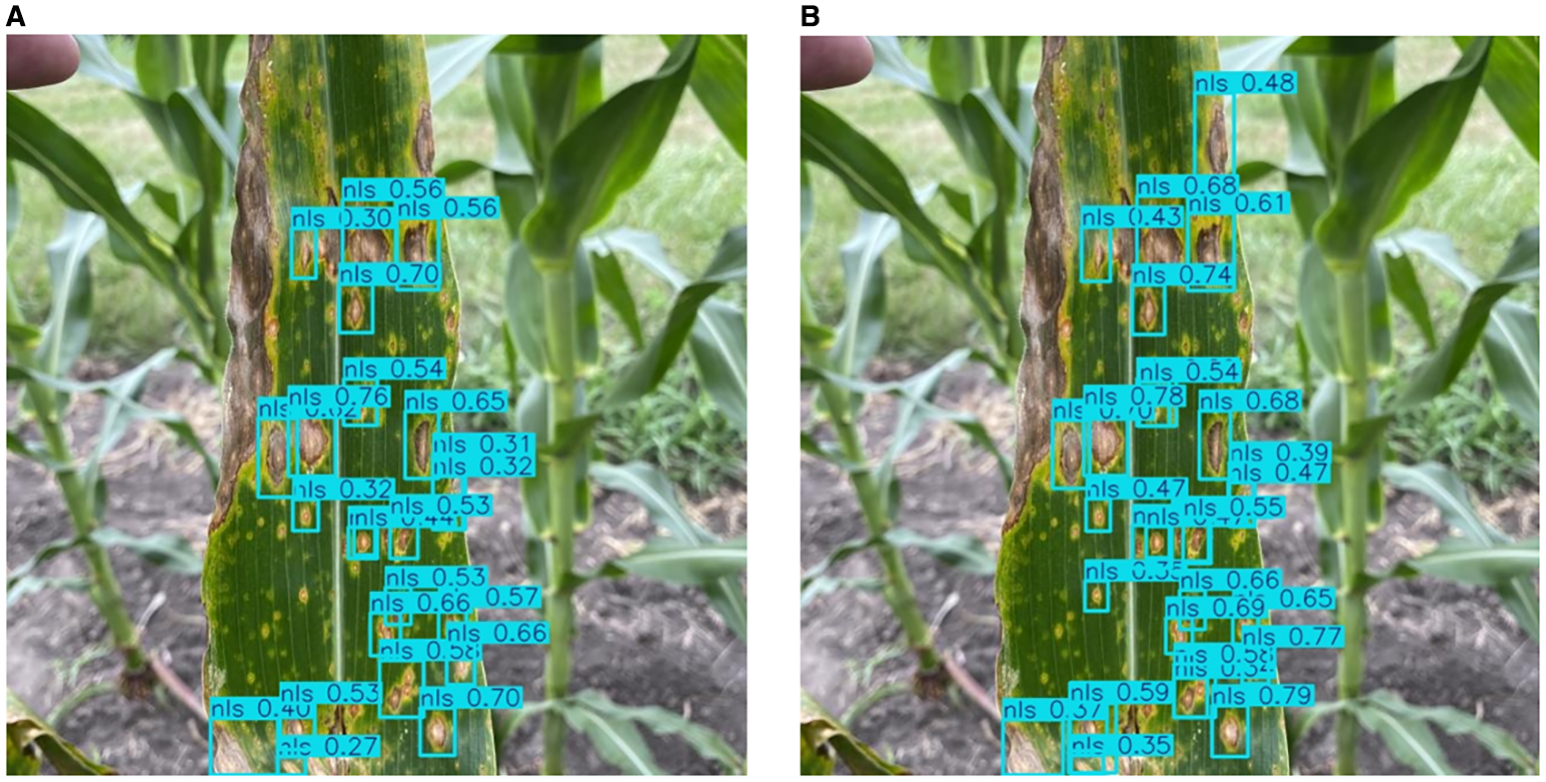
Detection results from the baseline YOLOv11n model **(a)** and the SEAFEC-enhanced model **(b)** on the same maize leaf image containing dense and small lesions. Bounding boxes indicate predicted Northern Leaf Spot (NLS) regions, illustrating the difference in small-object detection performance between the two models.

As shown in **Figure 9**,when detecting dense and small Northern Leaf Spot (NLS) lesions, the baseline model fails to capture many targets, resulting in large areas of missed detection. In contrast, the SEAFEC-enhanced model successfully detects most of the missed instances. This visualization clearly confirms SEAFEC’s strong capability in modeling fine-grained spatial structures and indistinct boundaries, addressing the challenge of small-object detection in complex agricultural environments.

### 3.4 Weed segmentation experiments

To comprehensively evaluate the ability of the proposed modules to address fine-grained weed segmentation challenges, we applied them to the sugarcane–weed dataset provided by Sun et al (Sun et al., 2024). Weed segmentation is critical for distinguishing crops from weeds and serves as the foundation for precision weeding. The dataset was collected under real-world field conditions at the experimental fields of Guangxi University’s Institute of Agro-Industry Development. To ensure diversity, the images were captured across multiple time periods, under different weather conditions, and during various growth stages of sugarcane, often featuring uneven illumination, occlusion, and intertwined crop–weed structures. The original images have a resolution of 6016 × 4016 pixels, which were processed into 1024 × 1024 patches using overlapping cropping to improve computational efficiency, resulting in a total of 23,100 images. All images were manually annotated at the pixel level into three categories: background, sugarcane, and weeds, represented by pseudo-colors black, red, and green, respectively, providing a rigorous benchmark for evaluating the ability of SEAFEC to achieve precise boundary delineation in crop–weed discrimination.

Compared with classification and detection, this dataset presents more significant challenges for segmentation, as sugarcane and weeds often exhibit similar appearances, irregular contours, and frequent overlaps. This requires models not only to classify pixels correctly but also to delineate precise boundaries at a fine structural level. For training and evaluation, the dataset was split into 60% training, 20% validation, and 20% testing sets, ensuring reliable model optimization and fair generalization assessment.

For the segmentation task, performance is evaluated using mean IoU (mIoU) for each object category and overall, mean Precision, and mean Recall. mIoU measures the average overlap between predicted and ground-truth regions, mean Precision reflects the proportion of correctly predicted pixels among all predicted pixels, and mean Recall indicates the proportion of correctly predicted pixels among all ground-truth pixels.

**Figure 10:**
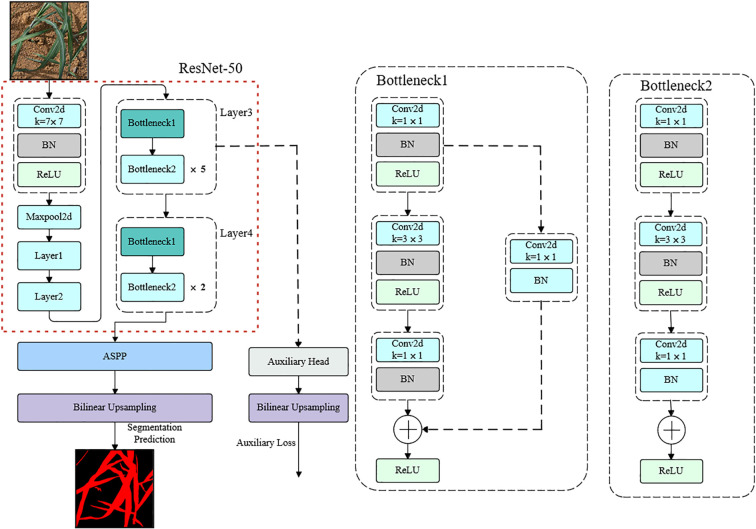
Overview of the DeepLabv3 architecture used in this study.

We used the classical DeepLabv3 framework with a ResNet-50 backbone as the baseline. As shown in **Figure 10**, the proposed modules were embedded into the deep bottleneck layers of ResNet-50 to enhance high-level semantic feature extraction.

The model was trained for 500 epochs using the SGD optimizer with a momentum of 0.9, weight decay of 1×10^-^^4^, and an initial learning rate of 0.001 following a scheduler with warmup. The input crop resolution was set to 480×480, and multi-scale training was applied (via RandomResize) to enhance robustness. The batch size was 8. A random seed was not explicitly specified in this training configuration.

In the SEAFEC-integrated variant, the fusion coefficient α was initialized to 0.4 and optimized during training, while the MEFE pooling kernel sizes were set to (Szegedy et al., 2014; Ren et al., 2015; Kamilaris and Prenafeta-Boldú, 2018) to effectively capture multi-scale edge features in the sugarcane–weed dataset.

Compared to classification and detection, semantic segmentation demands higher pixel-level precision, making boundary enhancement particularly crucial. In the test set, the edge-focused MEFE module became the primary contributor to performance improvement, yielding a notable mIoU gain of +2.3%, significantly higher than the +0.9% provided by SCARF. This is mainly because segmentation metrics such as mIoU are highly sensitive to boundary accuracy—any misclassification, blur, or disconnection at the crop–weed boundary directly affects performance. MEFE’s multi-scale edge enhancement mechanism effectively reinforces high-frequency details along object contours, enabling the model to clearly distinguish between intertwined plant structures that are otherwise visually ambiguous.

In contrast, SCARF plays a more supportive role by providing stable contextual understanding. Its adaptive receptive field allows the model to fully capture large-scale target regions, preventing fragmentation or missing areas in the prediction mask, thereby offering a consistent semantic foundation for MEFE’s refinement.

**Figure 11:**
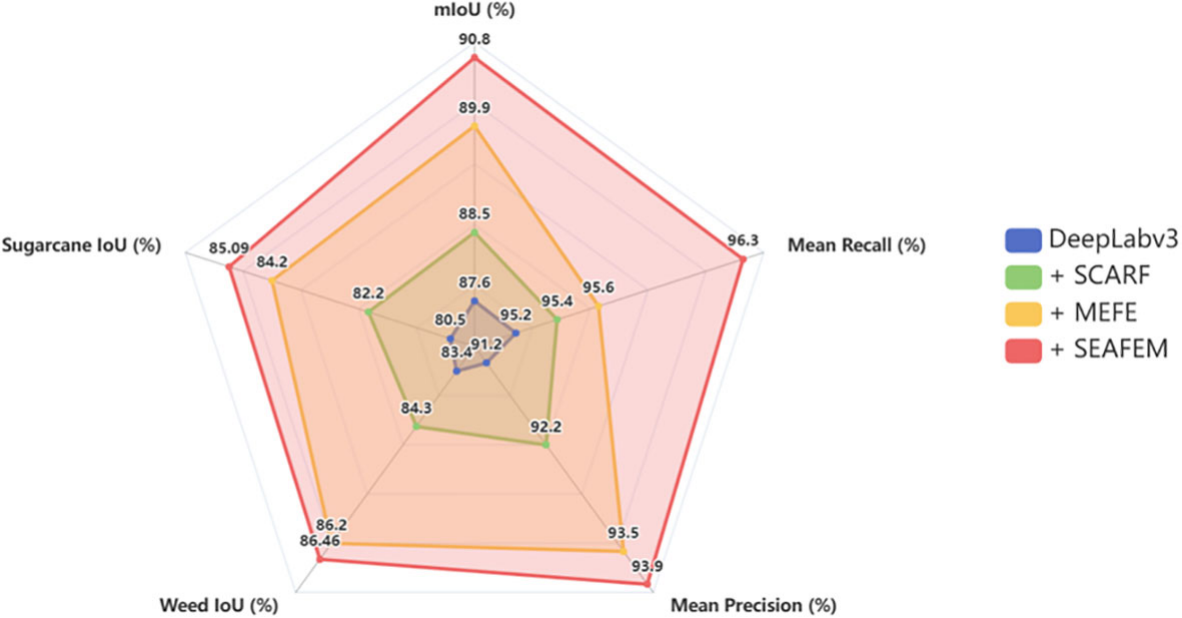
Multi-metric performance comparison of different models on the weed segmentation test set.

Overall, the combined SEAFEC module achieved the best performance, with an mIoU of 90.8% on the test set, surpassing all other configurations. As illustrated in the radar chart (**Figure 11**), SEAFEC led across all evaluation metrics, and particularly in class-specific IoU for “sugarcane” and “weed,” it achieved substantial gains of +4.59 and +3.26 percentage points, respectively.

**Figure 12:**
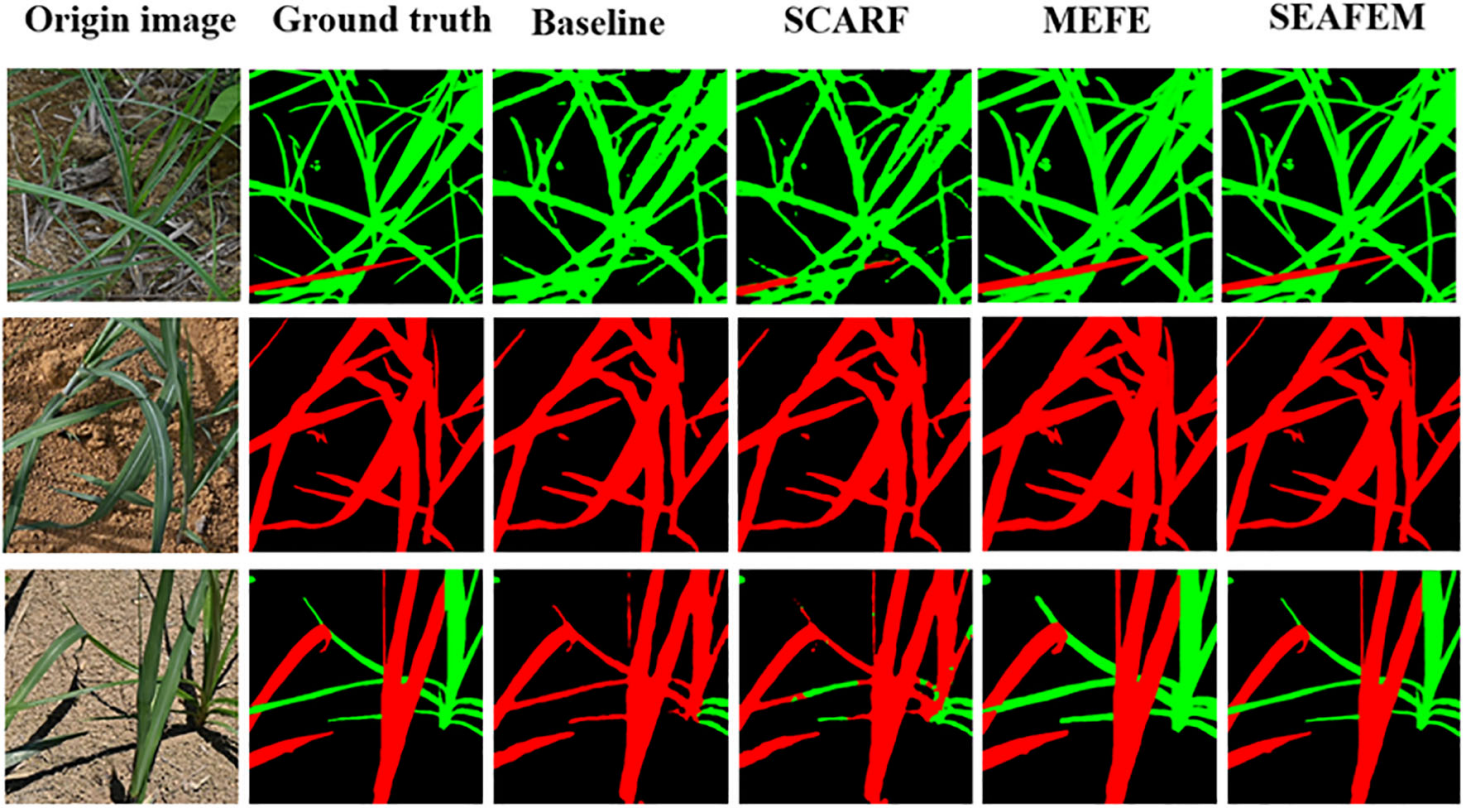
Qualitative analysis of different models on the weed segmentation task.

As shown in **Figure 12**, the segmentation results evolve from the baseline’s “blurred and entangled boundaries,” to SCARF’s “structural recovery,” and further to MEFE’s “edge sharpening.” SEAFEC delivers highly accurate pixel-level delineation of fine weeds and overlapping leaves. These results strongly validate the effectiveness of the proposed synergistic enhancement strategy in addressing edge-challenging segmentation tasks under complex agricultural conditions.

As shown in **Table 6**, integrating the proposed modules into DeepLabV3 leads to different impacts on model complexity. SCARF introduces only a negligible increase in both parameters (+0.6%) and FLOPs (+5.0%), while MEFE moderately increases parameters (+4.2%) but achieves a substantial reduction in FLOPs (−25.0%). In contrast, SEAFEC significantly increases both parameters (+24.8%) and FLOPs (+15.0%).

**Table 6:** Parameter and FLOPs variations of DeepLabV3 with different modules integrated.

Model	Params(M)	ΔParams(M)	FLOPs(G)	ΔFLOPs(G)
DeepLabV3	39.63	—	22.04	—
+SCARF	39.89	**+0.6%**	23.14	+5.0%
+MEFE	41.29	+4.2%	16.53	**-25.00%**
+SEAFEC	49.48	+24.8%	25.35	+15.0%

The bold values indicate the best performance (highest metric) in each column.

The underlying reasons for these changes are consistent with those observed in the classification experiments: SCARF adds lightweight spatial–channel attention with minimal overhead; MEFE reduces computational cost through grouped depthwise convolutions despite introducing extra branches; and SEAFEC’s dual-branch fusion inflates channel dimensions, resulting in higher complexity. Compared with the classification setting, the relative growth ratios of parameters and FLOPs in segmentation are smaller. This is mainly because DeepLabV3 itself has a much larger parameter base, which dilutes the relative increase brought by the modules. Nevertheless, the modules remain computationally efficient given the substantial performance gains.

### 3.5 Failure case analysis

Although SEAFEC demonstrated significant performance advantages across the three main tasks, we also identified several typical failure cases during the experiments. These ‘hard examples’ expose the limitations of SEAFEC when dealing with extreme visual challenges and clearly point the way for our future improvements.

**Figure 13:**
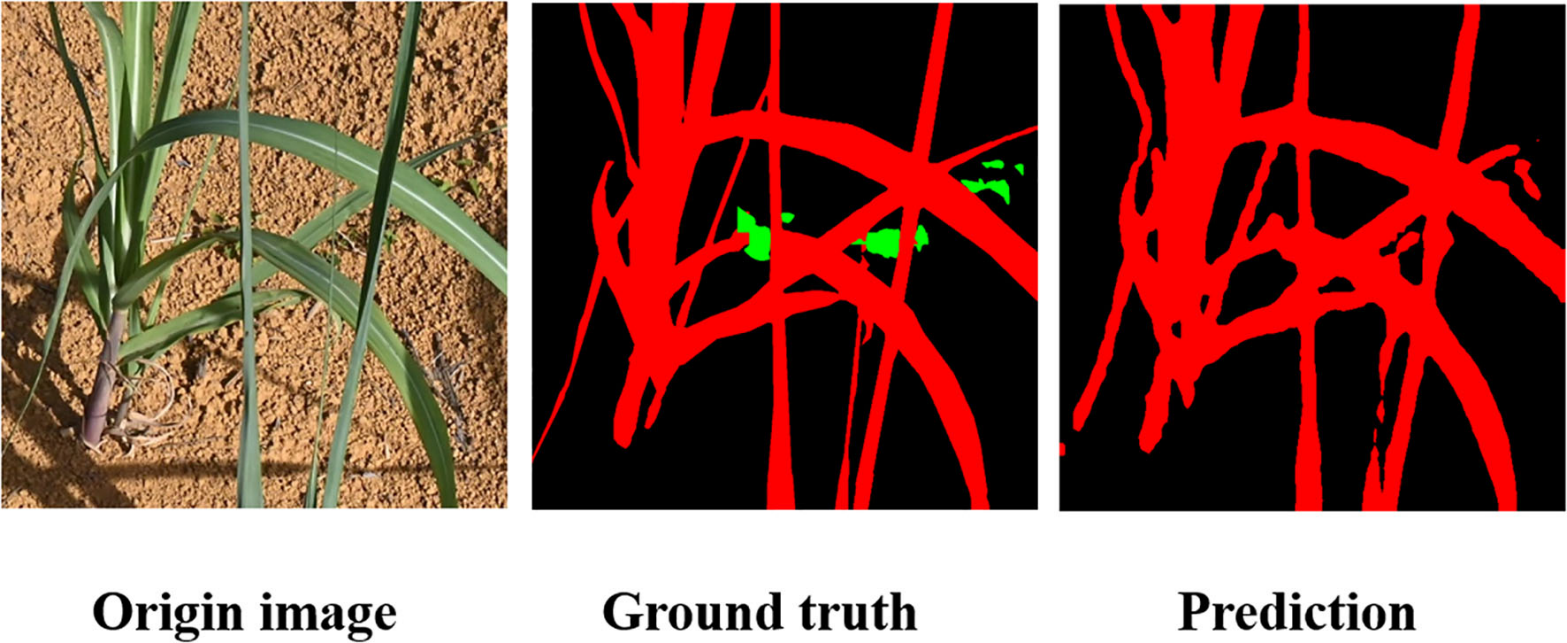
Feature confusion failures in weed segmentation.

For instance, in the weed segmentation task, we observed a representative failure pattern, as shown in **Figure 13**, which reveals a key limitation of SEAFEC when handling targets that are highly similar and spatially intertwined. In the weed segmentation dataset, the “background” and “sugarcane” classes (red regions in this example) dominate the pixel distribution, while the “weed” class (green) occupies only a small fraction, representing a severe class imbalance. The weeds are often occluded by sugarcane leaves and visually blended with the soil background, making their boundary cues extremely weak. Although SEAFEC achieved a 3.4% improvement in mIoU and significantly enhanced boundary localization, it still struggles with these extreme “hard examples” characterized by severe imbalance and high inter-class similarity. In such cases, the edge cues between “weed–sugarcane” or “weed–soil” are too subtle, while the “sugarcane–soil” boundary dominates the edge response. As a result, the MEFE branch may learn to emphasize stronger edges while neglecting weak, small-scale weed boundaries. Similarly, SCARF, which adaptively adjusts its receptive fields, may focus more on the dominant sugarcane regions, unintentionally suppressing the faint responses from minor weed areas during contextual aggregation. Future improvements will aim to address these issues. One direction involves optimizing the MEFE module; the current MEFE design employs fixed multi-scale pooling kernels (e.g (Szegedy et al., 2014; Simonyan and Zisserman, 2015; Kamilaris and Prenafeta-Boldú, 2018).,), so future work could explore more flexible designs, such as introducing dilated convolutions or smaller-scale pooling to construct a dedicated “micro-object” branch that enhances sensitivity to fine-grained details. Another key direction is integrating Transformer architectures. As noted in the limitations, SEAFEC has so far been validated only within CNN frameworks. Future extensions may combine SEAFEC with lightweight Transformer backbones (e.g., MobileViT or Swin Transformer). The global attention mechanism of Transformers can effectively capture sparse and isolated small-object features, while SEAFEC’s edge enhancement (MEFE) can complement Transformers by recovering fine structural details—potentially forming a stronger hybrid architecture for complex agricultural imagery.

**Figure 14:**
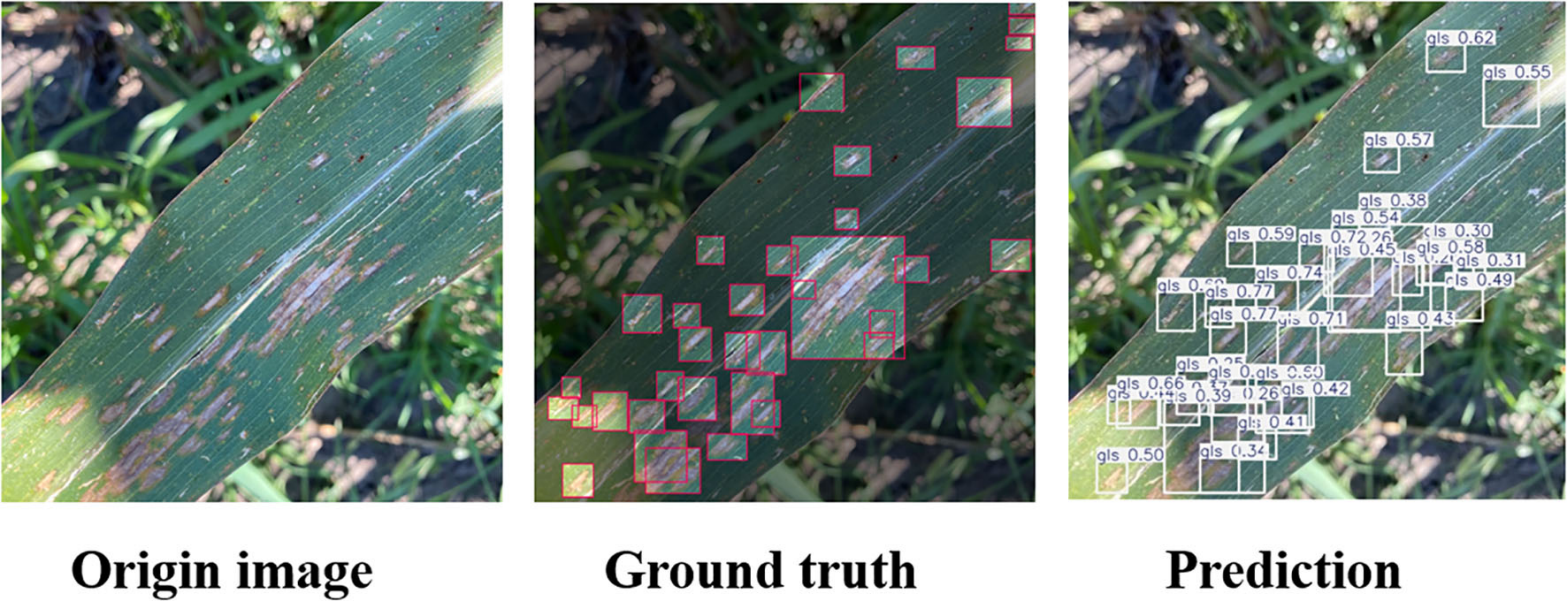
Corn disease detection failures under dense conditions.

As illustrated in **Figure 14**, this case highlights the limitations of SEAFEC when confronted with the dense and small-object challenge in the CD&S dataset. The model exhibits two opposite types of errors—over-detection in dense regions and missed detection at leaf boundaries. In the dense lesion area near the leaf center, the model produces far more detection boxes than the ground-truth annotations. For instance, a single large lesion in the annotation is fragmented into multiple overlapping boxes with similar confidence scores. This phenomenon can be attributed to two main factors. The first is a side effect of the MEFE module; MEFE is designed to enhance edge and texture details, but in highly textured or overlapping lesion regions, it may also amplify subtle intra-lesion variations or blurred inter-lesion boundaries. This excessive edge enhancement can cause the detector to split a semantically large lesion into several smaller ones. The second factor is the failure of Non-Maximum Suppression (NMS); the model outputs multiple overlapping boxes with similar confidence values, making it difficult for standard NMS to retain only the best one. This indicates instability in the bounding-box regression under dense conditions, where redundant detections rely heavily on post-processing to be suppressed.

Conversely, at the outer leaf margins, the model fails to detect true lesions. This opposite error pattern is mainly caused by context contamination. This points to a limitation of SCARF, which generates dynamic receptive fields by aggregating contextual information via average pooling. For central lesions, the context is dominated by healthy leaf tissue; but for edge lesions, the surrounding context includes both leaf and complex background (e.g., soil or other plants). Such “mixed” context may distort attention generation, leading to weakened or incorrect spatial weighting and thus missed detections. This also reveals a limitation of MEFE, which enhances boundaries by computing the difference between feature maps and their smoothed versions. However, the boundary between “lesion–background” differs substantially from that between “lesion–leaf.” MEFE may fail to effectively enhance such boundary-on-boundary structures, resulting in weak feature activation and missed predictions.

Overall, this case demonstrates that SEAFEC’s robustness decreases under extremely dense and edge-localized lesion conditions. MEFE tends to “over-enhance” densely packed textures, leading to false positives and fragmentation, while both SCARF and MEFE struggle to maintain stable responses at leaf boundaries due to inconsistent contextual cues. Future work will focus on improving feature normalization and adaptive context modeling to enhance detection stability in dense agricultural scenes.

### 3.6 Discussion

The proposed SEAFEC module directly addresses two fundamental challenges that frequently hinder reliable crop monitoring: multi-scale variability and blurred boundaries in plant disease and weed imagery. Across three representative tasks, SEAFEC demonstrated consistent improvements. On the PlantVillage dataset (Hughes and Salathé, 2015), it improved disease classification accuracy by +1.8%, supporting more reliable identification of crop diseases at early stages. On the CD&S maize dataset (Ahmad et al., 2021), SEAFEC achieved a +2.5% mAP gain, highlighting its ability to localize lesions of diverse shapes and sizes under complex field conditions. For weed management, its strongest advantage was observed on the sugarcane–weed dataset (Sun et al., 2024), where SEAFEC reached 90.8% mIoU (+3.4% over DeepLabV3) and substantially improved class-specific IoU for “weed” (+3.26 percentage points), enabling precise delineation of crop–weed boundaries essential for precision weeding. Together, these results demonstrate that SEAFEC provides a unified solution for overcoming the scale–boundary dilemma in agricultural image analysis, thereby contributing to more accurate disease diagnosis and sustainable weed control. These consistent gains across classification, detection, and segmentation further highlight SEAFEC as a general-purpose solution for plant health monitoring tasks.

Compared with existing methods such as Deformable Convolution Networks (DCNs) (Zhu et al., 2019), which mainly enhance shape adaptivity, or RFAConv (Zhang et al., 2024), which focuses on receptive field adjustment, SEAFEC offers a more balanced approach by jointly enhancing scale adaptivity and boundary perception. This design choice directly benefits agricultural applications, where both global lesion context and fine edge details are equally critical for decision-making.

Despite these advantages, SEAFEC has two notable limitations. First, the dual-branch fusion increases parameter size and computation, which may limit its deployment in resource-constrained scenarios such as mobile devices or field robots. Second, the evaluation was restricted to a limited number of public datasets, which may not fully capture the variability of real-world agricultural environments, including diverse crop species, growth stages, and ecological conditions.

Moreover, the increase in parameters and computational cost varies across the three experimental settings. This inconsistency is primarily attributed to differences in backbone architecture and integration strategy. In the classification task, SEAFEC was deeply embedded into the lightweight ResNet-18, where most standard convolutions in the later stages were replaced by SCARF and MEFE, leading to a relatively larger proportional increase in parameters. In contrast, the YOLOv11n-based detection experiment replaced only two convolutional layers in the backbone and neck, resulting in negligible changes in complexity. For segmentation, SEAFEC was selectively inserted into the ASPP branches of DeepLabv3, causing a moderate increase in computational cost while enhancing boundary sensitivity.

Such extensions will not only improve efficiency but also strengthen the general-purpose nature of SEAFEC, enabling it to adapt to broader agricultural applications. Future work will focus on two directions. Methodologically, SEAFEC can be extended to Transformer-based architectures (e.g., MobileViT, Swin Transformer) and lightweight CNNs (e.g., MobileNet) to combine global context modeling with high efficiency. From an agricultural perspective, further validation on large-scale, multi-crop, and multi-region datasets is essential to enhance robustness and support cross-crop generalization. Such extensions will enable SEAFEC to better serve practical applications in precision agriculture, supporting sustainable crop production through reliable disease monitoring and targeted weed management.

## 4 Conclusions

In this work, we addressed the critical challenges of scale variation and boundary ambiguity in plant disease and weed imagery. To overcome these limitations, we proposed SEAFEC, a novel convolutional module that integrates SCARF for dynamic receptive field adjustment and MEFE for multi-scale edge enhancement. This dual-branch design enables SEAFEC to capture both structural diversity and fine-grained boundary cues, which are often overlooked by conventional convolutional operators.

Extensive experiments on three representative tasks—disease classification, disease detection, and weed segmentation—demonstrated that SEAFEC consistently achieves highly effective performance across different recognition paradigms. These results confirm that SEAFEC is not only effective in enhancing feature representation but also general-purpose, showing broad applicability in plant health management scenarios ranging from early disease diagnosis to precision weeding.

Looking forward, SEAFEC can be further extended to other crops, disease types, and field environments, offering a promising tool for the development of smart agriculture systems that integrate computer vision with crop protection practices. By offering both efficiency and generality, SEAFEC can serve as a practical building block for future agricultural vision systems.

## Data Availability

The original contributions presented in the study are included in the article/supplementary material. Further inquiries can be directed to the corresponding author.

## References

[B1] Ahmad A. Saraswat D. Gamal A. E. Johal G. (2021). CD&S dataset: handheld imagery dataset acquired under field conditions for corn disease identification and severity estimation. arXiv 2110.12084. doi: 10.48550/arXiv.2110.12084

[B2] Badrinarayanan V. Kendall A. Cipolla R. (2017). SegNet: A deep convolutional encoder-decoder architecture for image segmentation. IEEE Trans. Pattern Anal. Mach. Intelligence. 39, 2481–2495. doi: 10.1109/TPAMI.2016.2644615, PMID: 28060704

[B3] Chen Y. Dai X. Liu M. Chen D. Yuan L. Liu Z. (2020). “ Dynamic convolution: attention over convolution kernels,” in Proceedings of the IEEE/CVF Conference on Computer Vision and Pattern Recognition (CVPR). (Seattle, WA, USA: IEEE), 11030–11039.

[B4] Chen L. C. Papandreou G. Kokkinos I. Murphy K. Yuille A. L. (2018). DeepLab: semantic image segmentation with deep convolutional nets, atrous convolution, and fully connected CRFs. IEEE Trans. Pattern Anal. Mach. Intelligence. 40, 834–848. doi: 10.1109/TPAMI.2017.2699184, PMID: 28463186

[B5] Food and Agriculture Organization of the United Nations (2021). Scientific review of the impact of climate change on plant pests (Rome: FAO).

[B6] He K. Zhang X. Ren S. Sun J. (2015). “ Deep residual learning for image recognition” in Proceedings of the IEEE Conference on Computer Vision and Pattern Recognition (CVPR), 2016, 770–778. doi: 10.1109/CVPR.2016.90

[B7] Hughes D. P. Salathé M. (2015). An open access repository of images on plant health to enable the development of mobile disease diagnostics through machine learning and crowdsourcing. arXiv 1511.08060. doi: 10.48550/arXiv.1511.08060

[B8] Kamilaris A. Prenafeta-Boldú F. X. (2018). Deep learning in agriculture: A survey. Comput. Electron. Agriculture. 147, 70–90. doi: 10.1016/j.compag.2018.02.016

[B9] Krizhevsky A. Sutskever I. Hinton G. E. (2017). ImageNet classification with deep convolutional neural networks. Commun. ACM. 60, 84–90. doi: 10.1145/3065386

[B10] Lin M. Chen Q. Yan S. (2014) . Network in network. arXiv preprint arXiv:13124400. ( International Conference on Learning Representation (ICLR)). doi: 10.48550/arXiv.1312.4400

[B11] Liu W. Anguelov D. Erhan D. Szegedy C. Reed S. Fu C. Y. . (2016). “ SSD: single shot multiBox detector,” in Computer Vision – ECCV 2016. (Amsterdam, The Netherlands: Springer), 21–37.

[B12] Long J. Shelhamer E. Darrell T. (2015). “ Fully convolutional networks for semantic segmentation,” in Proceedings of the IEEE Conference on Computer Vision and Pattern Recognition (CVPR). (Boston, MA, USA: IEEE), 3431–3440. 10.1109/TPAMI.2016.2572683 27244717

[B13] Oerke E. C. (2006). Crop losses to pests. J. Agric. Science. 144, 31–43. doi: 10.1017/S0021859605005708

[B14] Redmon J. Divvala S. Girshick R. Farhadi A. (2016). “ You only look once: unified, real-time object detection,” in Proceedings of the IEEE Conference on Computer Vision and Pattern Recognition (CVPR). (Las Vegas, NV, USA: IEEE), 779–788.

[B15] Ren S. He K. Girshick R. Sun J. (2015). “ Faster R-CNN: Towards Real-Time Object Detection with Region Proposal Networks,” in Advances in Neural Information Processing Systems, (Red Hook, NY, USA: Curran Associates, Inc.), 91–99. Available online at: https://arxiv.org/abs/1506.01497 (Accessed December 10, 2025)., PMID:

[B16] Ronneberger O. Fischer P. Brox T. (2015). “ U-net: convolutional networks for biomedical image segmentation,” in Medical Image Computing and Computer-Assisted Intervention – MICCAI 2015. (Munich, Germany: Springer), 234–241.

[B17] Selvaraju R. R. Cogswell M. Das A. Vedantam R. Parikh D. Batra D. (2017). “ Grad-CAM: visual explanations from deep networks via gradient-based localization,” in Proceedings of the IEEE International Conference on Computer Vision (ICCV) (Venice, Italy: IEEE), 618–626.

[B18] Simonyan K. Zisserman A. (2015). ” Very deep convolutional networks for large-scale image recognition” in International Conference on Learning Representations (ICLR). doi: 10.48550/arXiv.1409.1556

[B19] Sun C. Zhang M. Zhou M. Zhou X. (2024). An improved transformer network with multi-scale convolution for weed identification in sugarcane field. IEEE Access. 12, 31168–31181. doi: 10.1109/ACCESS.2024.3368911

[B20] Szegedy C. Liu W. Jia Y. Sermanet P. Reed S. Anguelov D. . (2014). ” Going deeper with convolutions” in Proceedings of the IEEE Conference on Computer Vision and Pattern Recognition (CVPR). 2015, 1–9. doi: 10.1109/CVPR.2015.7298594

[B21] Ultralytics (2022). ultralytics/yolov5: v7.0 - YOLOv5 SOTA Realtime Instance Segmentation. Available online at: https://github.com/ultralytics/yolov5 (Accessed December 10, 2025).

[B22] Ultralytics (2023). Ultralytics YOLOv8: Cutting-edge object detection models. Available online at: https://github.com/ultralytics/ultralytics (Accessed December 10, 2025).

[B23] Wang Q. Wu B. Zhu P. Li P. Zuo W. Hu Q. (2020). “ ECA-net: efficient channel attention for deep convolutional neural networks,” in Proceedings of the IEEE/CVF Conference on Computer Vision and Pattern Recognition (CVPR) (Seattle, WA, USA: IEEE), 11534–11542.

[B24] Zhang X. Liu C. Yang D. Song T. Ye Y. Li K. . (2024). RFAConv: innovating spatial attention and standard convolutional operation. arXiv 2304.03198. doi: 2304.03198

[B25] Zhu X. Hu H. Lin S. Dai J. (2019). “ Deformable convNets v2: more deformable, better results,” in Proceedings of the IEEE/CVF Conference on Computer Vision and Pattern Recognition (CVPR). (Long Beach, CA, USA: IEEE).

